# Acute urinary tract infection elicits bladder afferent hypersensitivity

**DOI:** 10.1016/j.bbih.2025.100944

**Published:** 2025-01-15

**Authors:** Harman Sharma, Sarah K. Manning, Natalie E. Stevens, Georgia Bourlotos, Feargal J. Ryan, Cindy Tay, Sonja Klebe, Geraint B. Rogers, David J. Lynn, Steven L. Taylor, Luke Grundy

**Affiliations:** a Flinders Health and Medical Research Institute, Flinders University, Bedford Park, SA, Australia; b Microbiome and Host Health Programme, South Australian Health and Medical Research Institute (SAHMRI), Adelaide, SA, Australia; c Precision Cancer Medicine Theme, South Australian Health and Medical Research Institute (SAHMRI), Adelaide, SA, Australia; d Department of Anatomical Pathology, College of Medicine and Public Health, Flinders Health and Medical Research Institute, Flinders University, Adelaide, SA, 5042, Australia

**Keywords:** Urinary tract infection, Uropathogenic *E. coli*, Bladder, Afferent, Cystitis, Lower urinary tract symptoms

## Abstract

• We explored the neurophysiology underlying painful bladder sensations during UTI.
• UTI induces significant bladder afferent hypersensitivity during distension.
• Low-threshold afferents elicit exaggerated responses at normal bladder pressures.
• Afferent hypersensitivity correlated with the development of bladder dysfunction.
• Bladder afferents are key regulators of sensory and behavioural responses to UTI.

## 1 Introduction

Urinary tract infections (UTIs) are one of the most common bacterial infections globally, with over 400 million cases annually (Yang et al., 2022). Acute UTI can trigger a variety of distinctive bladder sensations, including dysuria, urinary urgency, irritation, and pelvic pain that disrupt the normal rhythm of periodic micturition and provoke rapid and frequent bladder voiding (Yang et al., 2022; Hooton, 2012). Despite this, the neurophysiology underlying aberrant bladder sensation and altered bladder function during UTI have yet to be determined.

Bladder sensations originate in the periphery, via the activation of sensory (afferent) nerves embedded throughout the bladder wall (Grundy et al.). Mechanosensory bladder afferents are activated during bladder distension and sensory signals feed into central nervous system (CNS) pathways that initiate bladder sensations and ultimately regulate bladder function (de Groat and Yoshimura, 2009; Fowler et al., 2008). During inflammation, pro-inflammatory mediators can disrupt normal bladder afferent function, causing them to become hypersensitive, a process known as peripheral sensitisation, that underpins the development of pain (de Groat and Yoshimura, 2009; Grundy et al., 2018a; Hucho and Levine, 2007; Wyndaele et al., 2024). Referred pelvic pain is a key feature of UTI in animal models (Montalbetti et al., 2022; Rosen and Klumpp, 2014) and hyperexcitability of bladder afferent pathways is a crucial mechanism underlying the development of the exaggerated sensations that define multiple inflammatory bladder disorders (Grundy et al.; de Groat and Yoshimura, 2009; Fowler et al., 2008; Grundy et al., 2018a; Wyndaele et al., 2024; Montalbetti et al., 2022). However, whether bladder afferent hypersensitivity to bladder distension develops during UTI has yet to be determined.

The aim of this study was to characterise the impact of UTI on bladder afferent signalling and bladder function using a preclinical model. We report that UTI induces hypersensitivity of low-threshold mechanosensitive afferents during bladder distension and the development of altered voiding patterns indicative of bladder dysfunction.

## 2 Methods

### 2.1 Animals

For this study, 8–10 weeks old female C57BL/6J mice were used following approval by the Animal Welfare Committee of Flinders University of South Australia (animal ethics #969-19). Due to the need for urethral access, only female mice were used in this study. Mice were group housed (5/cage) within the College of Medicine & Public Health Animal Facility (COMPAF) in ventilated cages, filled with corn cob bedding and shredded paper as well as free access to food and water, unless stated otherwise. Investigators were blinded to the treatment groups for all experiments.

### 2.2 Bladder infection

The uropathogenic *Escherichia coli* (UPEC) strain, CFT073 (700928 - ATCC), was used for all infection experiments. Frozen stock was streaked on Luria-Bertani (LB) agar and incubated overnight to obtain single colonies. Two single colonies were then mixed in LB broth and incubated overnight to a bacterial density of 1 × 10^9^ colony forming units (CFU)/ml, as determined by optical density spectrophotometry.

Under isoflurane anaesthesia, mice received an infusion of 40 μl of UPEC (1 × 10^9^ CFU/ml) or phosphate-buffered saline (PBS) (Sham control) into the bladder via transurethral catheterisation for 10min. 24hrs post infection, urine was collected from freely moving mice before being culled via CO_2_ asphyxiation prior to obtaining bladder tissue.

### 2.3 Bacterial load assessment

UPEC load from urine and bladder from Sham- and UPEC-infected mice was quantified by overnight culture on MacConkey agar (Thermo Fisher, #CM00078). MacConkey agar was used to selectively inhibit growth of non-UPEC organisms. Bladder and kidney tissue were first homogenised in PBS using a sterilised chrome bead, and all samples were serially diluted using LB broth with dilutions plated in triplicate. Plates were incubated overnight, and the average of dilutions with a countable colony number used to determine CFU/μl urine and CFU per bladder.

### 2.4 H&E

Bladders from mice treated with UPEC and Sham were horizontally bisected and were immediately placed into 4% paraformaldehyde (PFA) (Thermo Scientific, J61899 AK), then refrigerated at 2–8 °C. After 24hrs, bladders were transferred to 70% ethanol (Gibco) for 24hrs and then placed in a sucrose/PBS solution (0.01 M PBS + 30% sucrose + 0.01% sodium azide) and refrigerated overnight at 4 °C prior to processing within the Flinders University Microscopy and Microanalysis facility. Tissues were embedded with OCT and snap frozen under cryoprotection using cooled isopentane in liquid nitrogen. The frozen tissues were cut at 12 μm thickness and 6 serial sections were collected directly onto room temperature polyethyleneimine (PEI) coated slides and vacuum dried for 30min. Following a PBS wash, slides were stained with Haematoxylin & Eosin, dried, and imaged. Three full sections per bladder were scored for inflammation as per criteria outline in Table 1 – Supplementary materials.

### 2.5 Flow cytometry

#### 2.5.1 Immune cell isolation

Bladders were excised from UPEC and Sham treated mice and immediately placed into Roswell Park Memorial Institute medium (RPMI). For processing, bladders were bisected, and one half placed in digest buffer (1 mL) containing 1 mg/mL collagenase (Sigma-Aldrich #C9891), 100 μg/mL DNAse (Sigma-Aldrich #DN25) and 1% FCS (CellSera) in RPMI. Tissue was manually minced using fine dissecting scissors (WPI) before digestion for 1 hr (37°, 180RPM). Digestion was stopped by addition of 1 mL RPMI containing 10% FCS. Cells were filtered through a 70 μm cell strainer and centrifuged (400RCF, 5min) before red blood cell lysis (BD PharmLyse). Cells were washed in FACS buffer (0.1% BSA, 2 mM EDTA in PBS) and plated into 96 well round-bottom plates for antibody staining.

### 2.6 Flow cytometry and analysis

Cells were pelleted and resuspended in 50 μl prepared antibody cocktail (Table 2 – Supplementary materials). Following 30min incubation on ice, cells were washed with 200 μl FACS buffer, centrifuged (300 × *g* for 5min at 4 °C) and resuspended in 50 μL secondary antibody (Steptavidin-PE-CSF594; BD Biosciences) diluted 1:500 in FACS buffer. Cells were incubated for 15min and washed twice with 200 μl FACS buffer. DAPI stain (1/1000) was added to samples prior to acquisition. Data were acquired on a BD LSR Fortessa X-20 5 laser instrument and analysed using FlowJo v 10.8.1. The gating strategy used to identify individual immune cell populations is shown in Supplementary Fig. 5.

### 2.7 Voiding assay

Void Spot Assay (VSA) was performed as previously described (Grundy et al., 2018b) on mice 1-day after UPEC or Sham treatment. Mice were single housed for 1 week prior to VSA commencement to allow acclimation to single housing prior to VSA. For the assay, bedding was removed from the home cage and the bottom of the cage was lined with filter paper. Mice remained in their cages with free access to food and water for 3hrs. After 3hrs, filter paper was collected and stored for imaging.

Filter paper was imaged using a Bio-Rad ultraviolet trans-illuminator Universal Hood III (Biorad, ChemiDoc), then translated into a binary format using ImageJ software (NIH). The size and number of void spots was determined using thresholds in the ImageJ software. Urine spots were categorised into two distinct size ranges: small (spanning 500-100,000 pixels), and large (exceeding 100,000 pixels).

### 2.8 Ex-vivo whole bladder afferent recordings

Nerve recordings were performed using a previously described *ex vivo* model (N = 4/5 group) (Grundy et al., 2018b, 2020). Mice were humanely killed via CO_2_ inhalation, and the entire lower abdomen was removed and submerged in a modified organ bath under continual perfusion with gassed (95% O_2_ and 5% CO_2_) Krebs-bicarbonate solution at 35 °C. Ureters were tied with 4-0 perma-hand silk (Ethicon, Raritan, NJ, #LA53G). The bladder was catheterised (PE 50 tubing) via the urethra and connected to a syringe pump (NE-1000) to allow a controlled fill rate of 100 μL/min with saline (NaCl, 0.9%). A second catheter connected to a pressure transducer (NL108T2; Digitimer, United Kingdom) was inserted through the dome of the bladder and secured. Pelvic nerve fibers were dissected into fine multiunit branches, and a single branch was placed within a sealed glass pipette containing a microelectrode (WPI) attached to a Neurolog headstage (NL100AK; Digitimer). Nerve activity was amplified (NL104), filtered (NL 125/126, bandpass 50–5000 Hz, Neurolog; Digitimer), and digitised (CED 1401; Cambridge Electronic Design (CED), United Kingdom) to a PC for offline analysis using Spike2 software (CED). The number of action potentials was determined per second to quantify the afferent response to distension. Single-unit analysis was performed offline using Spike2 version 5.18 software and were characterised as low- and high-threshold as previously described (Brierley et al., 2020). “Low threshold” afferents exhibit continuous action potential firing at less than 16 mm Hg. “High-threshold” afferents exhibit continuous action potential firing only when pressures exceed 16 mm Hg.

## 3 Results

**Fig. 1:**
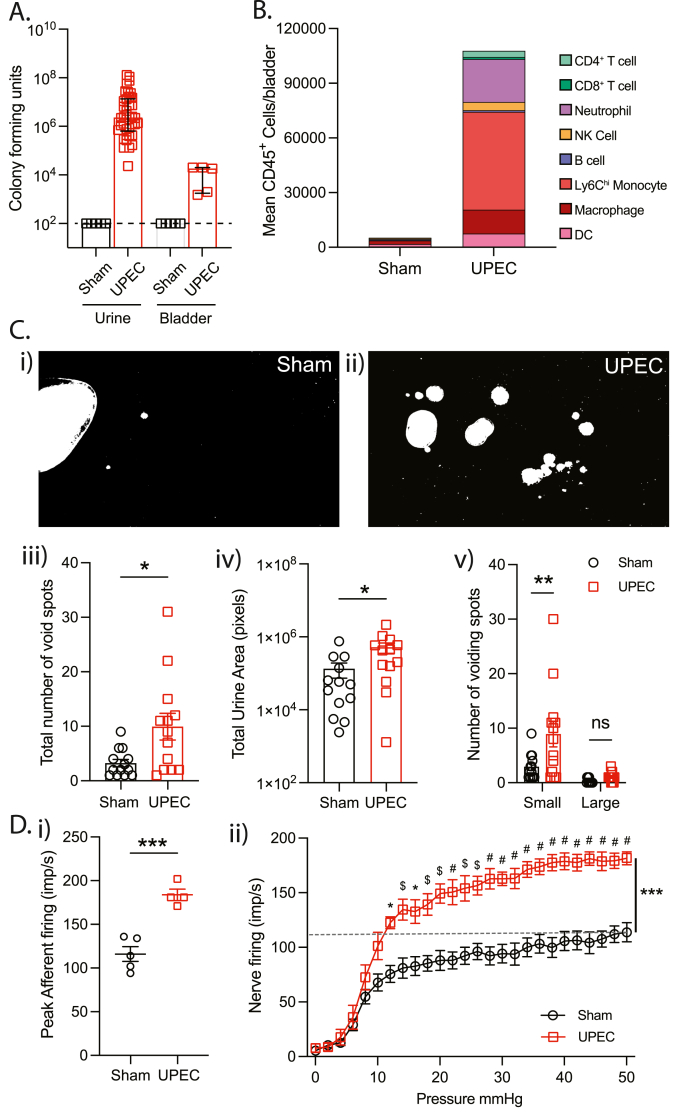
UPEC infection alters bladder voiding patterns and increases bladder afferent mechanosensitivity to distension. (A) Bacteria was detected in the urine (CFU/μl) and bladders (CFU/bladder) of mice 24hrs after UPEC instillation but not 1X PBS (Sham) into the bladder (n = 5–30). Black dash line represents limit of detection. (B) Immune cell phenotyping from bladders of sham (N = 8) and UPEC-treated (N = 5) mice revealed a significant increase in CD45^+^ cells in bladders from UPEC mice compared to Sham. Bladder function in sham and UPEC mice was assessed by void spot assay over 3hrs (C). (Ci) UPEC mice exhibit a significant increase in the total number of void spots compared to Sham (N = 13, ∗p < 0.05, unpaired *t*-test), a significant increase in small sized urine spots based on pixel density (small; 500-100,000, large; >100,000 pixels (Cii) (N = 13, ∗∗p < 0.01, two-way ANOVA), and (Ciii) an increase in total urine area (N = 13, ∗p < 0.05, unpaired *t*-test). (Civ-v) Representative examples of filter paper used to collect urine from Sham and UPEC mice following visualisation with a UV transilluminator. (D*) Ex vivo* bladder nerve recordings were performed during bladder distension (0–50 mmHg) with saline in sham and UPEC mice. (Di) Peak mechanosensitive afferent responses to distension are significantly increased during UPEC infection (N = 4–5, ∗∗∗p < 0.001, unpaired *t*-test). (Dii) Bladder afferents exhibit significant hypersensitivity to distension during bladder distension (N = 4–5 mice: Sham vs UPEC ∗∗∗P < 0.001, two-way ANOVA. Sidak multiple comparisons at each pressure: ∗P < 0.05, ^$^P < 0.01, ^#^P < 0.001). ANOVA – analysis of variance. Dash-line in Bii represents peak firing rate of afferents in Sham mice.

At 24hrs after inoculation, mice were assessed for bacterial load in urine and bladder tissue using culture-based enumeration (Fig. 1A), bladder immune cell infiltration via flow cytometry analysis (Fig. 1B, Supplementary Fig. 1), and tissue damage via histopathological scoring of bladder sections (Supplementary Fig. 2).

Inoculation with UPEC led to clinically relevant indicators of UTI at 24hrs post infusion (Hooton, 2012; Abraham and Miao, 2015), including bacterial colonisation of the urine and bladder wall (Fig. 1A) and a substantial inflammatory response characterised by significant increases in CD45^+^ cells in the bladder (Fig 1B, Supplementary Fig. 1Ai, Aii), including Neutrophils (Ly6G^+^), Inflammatory monocytes (MHCII^+^ F4/80^lo^ Ly6C^hi^), Dendritic cells (MHCII^+^ F4/80^-^ CD11c^+^), macrophages (MHCII^+^ F4/80^+^), helper (CD3^+^CD4^+^), and Cytotoxic (CD3^+^CD8^+^) T-cells, and NK (NK1.1^+^) cells (Fig 1B, Supplementary Fig. 1Aiii-ix). Histological assessment of bladder structure showed UPEC mice exhibited significant bladder damage, oedema, and haemorrhage compared to Sham mice (Supplementary Fig. 2A, B).

Bladder inoculation with UPEC also induced a clinical UTI phenotype, identified by the development of altered bladder function in awake, freely moving mice following UPEC infusion (Fig. 1C). Whilst Sham mice typically exhibited a voiding pattern with large, organised urine spots on the edge of the cage, UPEC mice developed altered voiding patterns (Fig. 1Ci, 1Cii), with a greater number of urine spots that were scattered throughout the cage (Fig. 1Ci, ii, iii) and a greater total urine spot area (Fig. 1Civ). Enumeration of urine spots based on size revealed significantly more small-sized (<100,000 pixels) spots from UPEC mice (Fig. 1Cv), indicating UPEC mice were voiding smaller volumes at shorter intervals, a behavioural phenotype indicative of bladder overactivity and UTI.

To determine whether acute UPEC infection evoked changes in bladder sensory signalling, intravesical pressure and mechanosensitive afferent firing were recorded during bladder distension (0–50 mmHg) using an established *ex vivo* bladder afferent nerve recording technique (Fig. 1D) (Grundy et al., 2018b). UPEC mice displayed a significantly exaggerated peak afferent response to bladder distension (Fig. 1Di). Furthermore, assessing afferent responses throughout bladder filling (0–50 mmHg) revealed significant afferent hypersensitivity to distension with signalling intensities observed only at noxious (50 mmHg) bladder pressures in Sham mice observed at physiological bladder pressures (<12 mmHg) in UPEC mice (Fig. 1Dii). Bladder afferent hypersensitivity occurred in the absence of changes in bladder compliance (Supplementary Fig. 3), indicating that changes in bladder afferent signalling were due to direct sensitisation of mechanosensory afferents rather than secondary to altered bladder muscle function.

**Fig. 2:**
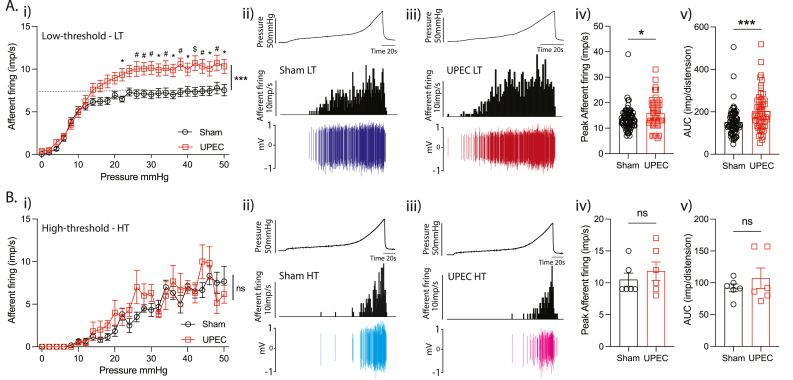
Low- but not high-threshold mechanosensitive bladder afferents are sensitised during UPEC infection. Based on activation threshold during bladder distension, bladder afferents were classified as either low-threshold (LT) or high-threshold (HT). (Ai) UPEC infection significantly enhances low-threshold (LT) mechanosensory responses to distension (Sham (n = 76) vs UTI (n = 51) ∗∗∗P < 0.001, two-way ANOVA. Sidak multiple comparisons at each pressure: ∗P < 0.05, ^#^P < 0.01, ^$^P < 0.001). Experimental trace of single LT mechanosensory afferents from Sham (Aii) and UPEC (Aiii) mice during bladder distension (0–50 mm Hg). (Aiv) Peak mechanosensory response to distension in LT afferents was significantly enhanced during UPEC infection (Sham (13.70 ± 0.49) vs UPEC (15.90 ± 0.81) ∗P < 0.05). (Av) Total area under the curve (AUC) of LT mechanosensory afferent response to distension was significantly enhanced during UPEC infection (Sham (151.8 ± 7.5) vs UPEC (204 ± 13.2) ∗∗∗P < 0.01). (Bi) UPEC infection had no impact on high-threshold (HT) mechanosensory responses to distension (Sham (n = 6) vs UPEC (n = 6) ^ns^ P > 0.05, two-way ANOVA. Sidak multiple comparisons at each pressure. Experimental trace of single HT mechanosensory afferents from Sham (Bii) and UPEC (Biii) mice during bladder distension (0–50 mm Hg). (Biv) Peak mechanosensory response to distension in HT afferents was unchanged during UPEC infection (Sham (10.5 ± 1.02) vs UPEC (11.83 ± 1.47) ^ns^P > 0.05). (Bv) Total area under the curve (AUC) of HT mechanosensory afferent response to distension was unchanged during UPEC infection (Sham (92 ± 6.11) vs UPEC (107 ± 16.01) ^ns^P > 0.05). ANOVA – analysis of variance. Dash-line in Ai represents peak firing rate of LT afferents in Sham mice.

To assess differences in individual mechanosensitive afferents, we performed post-hoc linear interpolation of the afferent signal from our multiunit recordings (Supplementary Fig. 4) and identified 139 distinct mechanosensitive afferent waveforms. Mirroring the effect we observed in our multiunit afferent recordings, single unit mechanosensitivity was found to be significantly greater in afferents from UPEC mice compared to Sham, with exaggerated total and peak afferent responses to distension (Supplementary Fig. 4, 4i, 4ii, 4iii). Subtyping mechanosensitive afferents based on activation thresholds allows for the analysis of low-threshold (LT) afferents, which are activated by physiologically relevant bladder pressures (Sup Fig. 4Aiv). LT afferents exhibited hypersensitivity to bladder distension following UPEC infection, including significantly higher peak and total area under the curve (AUC) responses (Fig. 2Ai, 2Aii, 2Aiii, 2Aiv). In contrast, high-threshold (HT) afferents, which are typically only activated by noxious distension pressures, were unaffected by UPEC infection (Fig. 2Bi, 2Bii, 2Biii, 2Biv). Together these data support the induction of exaggerated signalling of bladder afferents during bladder distension by UPEC infection.

## 4 Discussion

The bladder is innervated by a complex network of sensory afferents that terminate throughout the detrusor smooth muscle and underlying mucosa (Grundy et al.). In health, bladder afferents encode mechanical stretch and convey sensations of bladder fullness to the spinal cord and higher brain centres that are necessary to coordinate micturition (de Groat and Yoshimura, 2009; Fowler et al., 2008). During UTI, patients experience heightened bladder sensations including bladder irritation, urinary urgency, dysuria, and pelvic pain (Grundy et al.; Mansfield et al., 2022). These sensations serve as crucial indicators of bladder pathophysiology; providing a conscious awareness of infection, and the stimulus to override the normal rhythm of periodic urination to rapidly and frequently empty the bladder. This study shows, for the first time, that UTI in mice sensitises bladder afferents to distension, leading to exaggerated sensory signalling from the bladder at physiological pressures.

A previous study showed that isolated bladder-innervating dorsal root ganglion (DRG) neurons from UPEC infected mice exhibit hypersensitivity *in vitro* (Montalbetti et al., 2022). Our data complement and extend these findings to show that UTI sensitises low-threshold (LT) mechanosensitive afferents to bladder distension such that physiologically relevant levels of bladder filling during UTI can trigger afferent firing typically associated with noxious/painful levels of bladder stretch. Consequentially, as bladder afferent signals feed into central nervous system circuits that initiate bladder sensations (Grundy et al.; de Groat and Yoshimura, 2009; Fowler et al., 2008), our findings suggest that bladder afferent hypersensitivity may be an important mechanism contributing to adverse bladder sensations in patients with acute UTI.

Unravelling the precise mechanisms underlying the development of bladder afferent hypersensitivity during UTI remains challenging due to the dynamic biological response to UPEC infection that involves urothelial signalling, immune cell recruitment, and the release of various pro-inflammatory mediators including histamine, cytokines and chemokines, and neuropeptides (de Groat and Yoshimura, 2009; Grundy et al., 2020; Brierley et al., 2020). Supernatants from UPEC infected mice, as well as isolated bacterial virulence factors, and bacterial toxins have been shown to directly sensitise bladder-innervating DRG *in vitro* (Montalbetti et al., 2022). Furthermore, acute intraluminal instillation of supernatants from UPEC infected mice can sensitise high-threshold (HT) mechanosensitive afferents to distention *ex vivo* (Brierley et al., 2020); further supporting interactions between biological mediators present during infection and bladder-innervating sensory nerves that relay to the CNS. While major differences in the experimental design prevents direct comparisons between this prior study showing HT afferent hypersensitivity and our current study showing LT hypersensitivity, we hypothesise that the different experimental models used (bladder instillation of supernatant versus a UPEC model capable of disseminated infection) would impact the specific subtypes and proportion of nerves that are in contact with the sensitising stimulus, especially those in deeper layers of the bladder wall due to the barrier nature of the urothelium (Spencer et al., 2018). The present study more accurately captures the pathophysiology of UTI, allowing for the full combination of biological interactions throughout the bladder mucosa (Abraham and Miao, 2015).

Alongside bladder afferent hypersensitivity we found UTI in mice led to the development of increased urinary frequency, a principal clinical phenotype of UTI (Hooton, 2012). Bladder function is mediated by the intensity of the afferent signal transduced from the bladder, which is integrated into the central micturition circuits within the pontine nuclei (Fowler et al., 2008; Grundy et al., 2018a). Accordingly, our data show correlations between bladder afferent hypersensitivity and altered bladder function during UTI. These data align with previous studies showing UTI can impact bladder function (Montalbetti et al., 2022), and the well characterised contribution of bladder afferent hypersensitivity to the symptomology of other inflammatory bladder disorders (Grundy et al.; de Groat and Yoshimura, 2009; Fowler et al., 2008; Grundy et al., 2018a; Wyndaele et al., 2024; Montalbetti et al., 2022). The importance of this neurophysiological response to restoring bladder homeostasis during UTI is unclear. However, increased susceptibility to both initial and recurrent infections is seen in patients with reduced bladder sensory insight, such as those with neurogenic bladder, spinal cord injury, and diabetic neuropathy (Dinh et al., 2019; Wang et al., 2023). Therefore, bladder afferent hypersensitivity during UTI may represent a crucial component of the host-defence response to bladder infection, providing awareness of infection that initiates a protective behavioural response. However, further research is required to definitively link exaggerated bladder afferent firing to altered bladder sensation and function.

In conclusion, this study shows that distension sensitive bladder afferents are sensitised during UTI, leading to exaggerated signals destined for integration into the CNS circuits that regulate bladder sensation and function.

## CRediT authorship contribution statement

**Harman Sharma:** Writing – review & editing, Writing – original draft, Visualization, Methodology, Investigation, Formal analysis, Conceptualization. **Sarah K. Manning:** Writing – review & editing, Writing – original draft, Investigation. **Natalie E. Stevens:** Writing – review & editing, Methodology, Investigation, Formal analysis. **Georgia Bourlotos:** Writing – review & editing, Investigation. **Feargal J. Ryan:** Writing – review & editing, Methodology, Formal analysis, Conceptualization. **Cindy Tay:** Writing – review & editing, Methodology, Formal analysis. **Sonja Klebe:** Writing – review & editing, Resources, Formal analysis. **Geraint B. Rogers:** Writing – review & editing, Supervision, Resources, Conceptualization. **David J. Lynn:** Writing – review & editing, Supervision, Resources, Methodology, Conceptualization. **Steven L. Taylor:** Writing – review & editing, Writing – original draft, Visualization, Supervision, Resources, Methodology, Investigation, Funding acquisition, Formal analysis, Conceptualization. **Luke Grundy:** Writing – review & editing, Writing – original draft, Visualization, Supervision, Resources, Project administration, Methodology, Investigation, Funding acquisition, Formal analysis, Conceptualization.

## Funding

This work was supported by a 10.13039/501100022217Flinders Foundation Health Seed Grant. S.L.T and F.J.R were supported by 10.13039/501100000925NHMRC Investigator Grants (2008625 and 2017404, respectively).

## Declaration of competing interest

The authors declare the following financial interests/personal relationships which may be considered as potential competing interests: Dr Luke Grundy, Dr Steven Taylor reports financial support was provided by 10.13039/501100022217Flinders Foundation. If there are other authors, they declare that they have no known competing financial interests or personal relationships that could have appeared to influence the work reported in this paper.

## Data Availability

Data will be made available on request.
