# Supplementary material for: Acute urinary tract infection elicits bladder afferent hypersensitivity
Source: Brain Behav Immun Health. 2025 Jan 15;44:100944. doi: 10.1016/j.bbih.2025.100944 (PMC11788684; doi:10.1016/j.bbih.2025.100944)
Supplement: Multimedia component 1 [file mmc1.docx]

**Supplementary Materials**

**Supplementary figures:**

**Sup Fig 1**: **UPEC bladder infection evokes a significant immune response. (A)**. % of live cells identified as CD45^+^ was significantly elevated in UPEC mice compared to sham (**Ai**). Neutrophils (Ly6G^+^) (**Aii**), Inflammatory monocytes (MHCII^+^ F4/80^lo^ Ly6C^hi^) (**Aiii**), Dendritic cells (MHCII^+^ F4/80^-^ CD11c^+^) (**Aiv**) and macrophages (MHCII^+^ F4/80^+^) (**Av**) were all significantly elevated in bladders from UPEC mice compared to sham. Helper (CD3^+^CD4^+^) (**Avi**) and Cytotoxic (CD3^+^CD8^+^) (**Avii**) T-cells, and NK (NK1.1^+^) cells (**Aviii**) were also significantly elevated in UPEC treated mice. Gating strategy for pan immune cell panel on bladder tissue. Data presented as median ± IQR for CFU and mean ± SEM for flow cytometry. Flow cytometry data analysed by unpaired t-test, *p<0.05, **p<0.01.


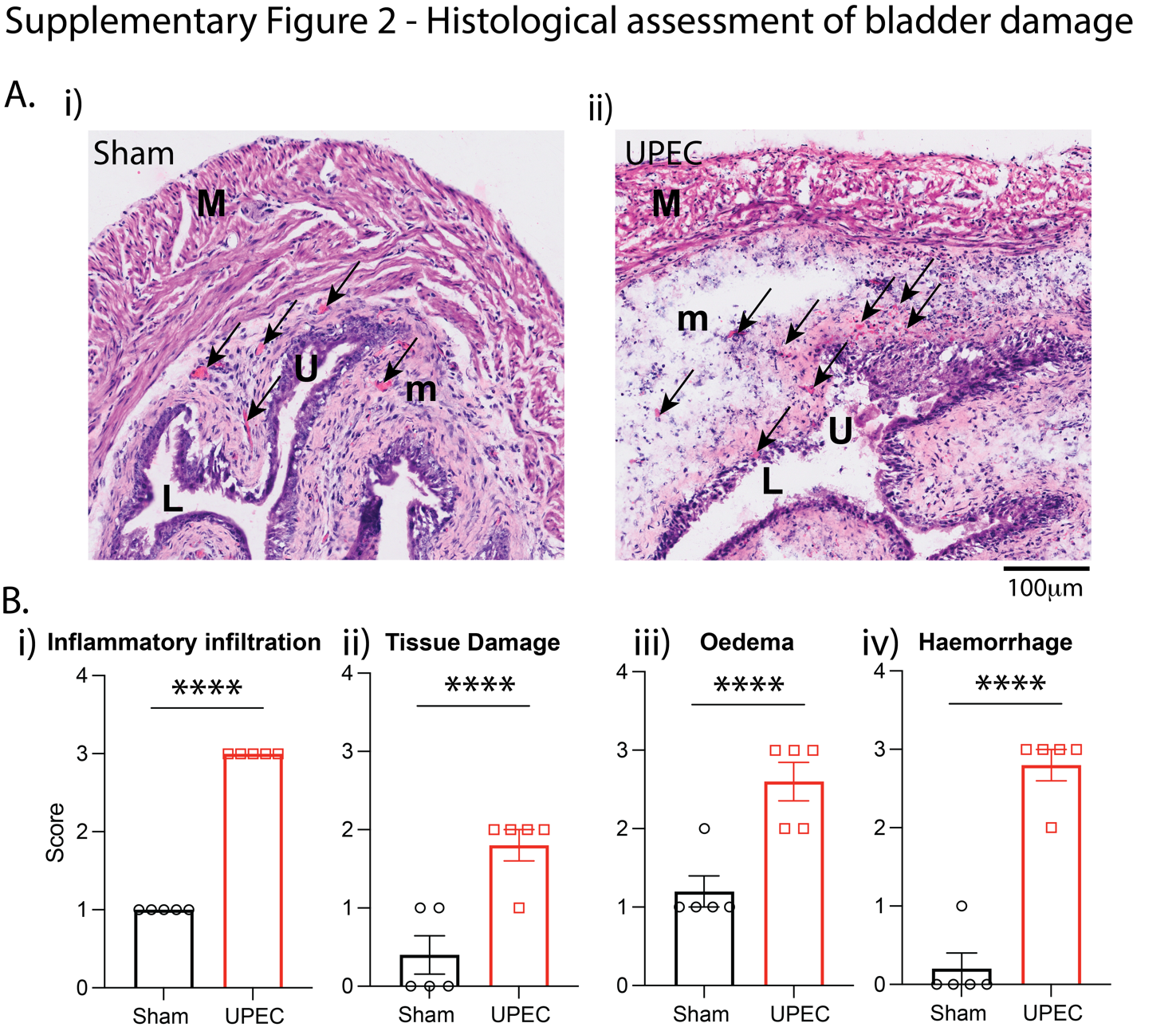


**Sup Fig 2**: **UPEC bladder infection evokes significant bladder damage**. **(A)** H&E-stained bladder sections exhibit increased immune cell infiltration (black arrows) and haemorrhagic regions, denoted by the presence of free red blood cells (black arrowheads), 24hrs after sham **(Ai)** or UPEC instillation **(Aii)**. **(B)** Histological quantification of bladder damage (using scoring criteria detailed in Table 1 of supplementary methods) demonstrates a significant increase in inflammatory infiltration **(Bi)**, tissue damage **(Bii)**, oedema **(Biii)** and haemorrhage **(Biv)** in bladders from UTI mice relative to Sham-treated bladders. L=lumen, m=mucosa, M=muscle. Scale bar=100mm. Data are presented as mean ± SEM and analysed by un-paired t-test (n=5/group, **** p<0.0001).

**Sup Fig 3**: **UPEC bladder infection has no impact on bladder compliance.** Graded distension of the bladder (0-50 mm Hg) in an ex vivo bladder-nerve recording preparation was performed with saline in sham and UPEC infected mice. UPEC infection had no significant impact on bladder muscle compliance (pressure/volume relationship) compared to sham mice. ^ns^P > 0.05, two-way ANOVA. Data are presented as mean ± SEM

**Sup Fig 4**: **UPEC bladder infection sensitises single unit bladder afferent mechano-sensitivity to bladder distension**. (**Ai**) UPEC infection significantly enhances single unit mechanosensory responses to distension (sham (n=82) vs UPEC (n=57) ***P < 0.001, two-way ANOVA. Sidak multiple comparisons at each pressure: *P < 0.05, ^#^P < 0.01). (**Aii**) Total area under the curve (AUC) of the mechanosensory afferent response to distension was significantly enhanced during UPEC infection **P < 0.01. (**Aiii**) Peak mechanosensory response to distension was significantly enhanced following UTI *P < 0.05. (**Aiv**) UPEC infection had no impact on the activation threshold of mechanosensitive bladder afferents ^ns^P > 0.05. Data are presented as mean ± SEM. Data in Aii, Aiii, Aiv analysed by unpaired t-test. HT- high threshold, LT – low threshold. Grey line in Aiv represents pressure cut-off for LT and HT afferent classification.

**Sup Figure 5**: Flow cytometry gating: Following gating for cells using forward scatter (FSC) and side-scatter (SSC) and single cells as depicted (**A**), autofluorescent urothelial cells were gated out using a combination of MHCII and SSC-W (**B**) and FSC-W (**C**) gates, as described in (1). Viable (**D**) and CD45^+^ (**E**) cells were selected, and lymphocytes (**F**) gated out using CD19/CD3 staining with CD11b staining used to separate autofluorescent myeloid cells. From the non-CD19/CD3^+^ population, neutrophils and NK cells were identified using Ly6G and NK1.1 staining, respectively (**G**). From remaining MHCII^+^ cells (**H**), F4/80+ bladder macrophages and Ly6Chi inflammatory monocytes were identified (**I**), and remaining cells gated for dendritic cells, identified by CD11c staining (**J**). From the CD19/CD3^+^ lymphocyte population, CD4 and CD8 were used to identify T cells (**K**), and B cells identified via MHCII staining (**L**)

Table 1: Histopathological scoring criteria for H&E bladder sections

| **Score** | **Inflammatory infiltration** | **Tissue damage** | **Oedema** | **Haemorrhage** |
| --- | --- | --- | --- | --- |
| 0 | No leukocytes | Full urothelium integrity | No oedema present | No free erythrocytes |
| 1 | <30 leukocytes | Sloughed urothelium (<25% of section) | Oedema limited to submucosa | <10 erythrocytes |
| 2 | 30-60 leukocytes | Sloughed urothelium (25-50% of section) | Oedema in the bladder wall, but not detrusor | 10-30 erythrocytes |
| 3 | >60 leukocytes | Sloughed urothelium (>50% of section) | Oedema present throughout the bladder wall | >30 erythrocytes |

Table 2: Pan Immune Cell Bladder Antibody Panel

| Target | Fluorophore | Dilution | Company | Cat# |
| --- | --- | --- | --- | --- |
| Fc Block | - | 1/400 | BD Biosciences | 553141 |
| CD45.2 | APC-Cy7 | 1/400 | BD Biosciences | 560694 |
| CD19 | PerCP-Cy5.5 | 1/200 | BD Biosciences | 551001 |
| CD3e | PerCP-Cy5.5 | 1/200 | BD Biosciences | 551163 |
| CD4 | BV510 | 1/200 | BD Biosciences | 563106 |
| CD8a | BUV395 | 1/200 | BD Biosciences | 563786 |
| NK-1.1 | APC | 1/200 | BD Biosciences | 550627 |
| Ly-6G | Pe-Cy7 | 1/400 | BD Biosciences | 560601 |
| CD11b | PE | 1/800 | BD Biosciences | 557397 |
| CD11c | Biotin | 1/50 | Miltenyi | 130-101-929 |
| MHCII (I-A/I-E) | BV711 | 1/1500 | BD Biosciences | 563414 |
| Ly-6C | FITC | 1/400 | BD Biosciences | 553104 |
| F4/80 | BV421 | 1/400 | BD Biosciences | 565411 |

**Supplementary references**

1. Mora-Bau G, Platt AM, van Rooijen N, Randolph GJ, Albert ML, Ingersoll MA. Macrophages Subvert Adaptive Immunity to Urinary Tract Infection. PLOS Pathogens. 2015;11(7):e1005044.
